# Genetic and morphological variation in the Colombian *Bombyx mori* germplasm: A first SSR-based assessment

**DOI:** 10.1371/journal.pone.0330183

**Published:** 2025-08-11

**Authors:** José Camilo González-Muñoz, Ana María López-Gutiérrez, Juan Carlos Sepúlveda-Arias

**Affiliations:** 1 Grupo Biodiversidad y Biotecnología, Facultad de Ciencias Ambientales, Universidad Tecnológica de Pereira, Pereira, Risaralda, Colombia; 2 Grupo Infección e Inmunidad, Facultad de Ciencias de la Salud, Universidad Tecnológica de Pereira, Pereira, Colombia; University of Waterloo, CANADA

## Abstract

Centuries of domestication have produced over 4000 *Bombyx mori* L. ecotypes, many of which share similar morphological and reproductive traits that hinder genetic improvement and differentiation. In Colombia, 67 silkworm lines are maintained; however, their industrial potential remains underutilized due to the lack of genetic and phenotypic data. To address this, we evaluated the genetic and morphological variability of the Colombian *B. mori* germplasm using 13 phenotypic traits and 23 simple sequence repeat (SSR) markers, 17 of which were polymorphic. Morphological traits such as voltinism (H’ = 4.22), larval markings (H’ = 4.057), and cocoon color (H’ = 3.849) were the most informative in differentiating breeds. The SSR markers revealed 2–11 alleles per locus, with PIC values ranging from 0.05 to 0.72 (median = 0.46). The AMOVA results revealed that 96% of the genetic variation occurred within populations, with only 4% among them. Commercial lines presented greater molecular homogeneity, likely due to intensive selective breeding, whereas ICA and unknown-origin (NC) lines presented no significant deviation from Hardy‒Weinberg equilibrium, suggesting greater genetic stability and potential for improving inbred breeds. Additionally, the molecular markers allowed for the classification of lines according to breeder-defined categories. Although several lines retained ancestral morphological characteristics, Japanese and Chinese lines presented low heterozygosity, likely due to bottlenecks associated with hybrid development programs. Morphological markers also revealed patterns on the basis of line provenance, offering clues to the origins of unclassified strains. A few lines clustered similarly across both the morphological and molecular dendrograms, indicating conserved trait patterns. The combined use of morphological and molecular markers allowed for a more comprehensive classification of the silkworm germplasm, identifying candidate lines for future breeding efforts. These findings provide new insights into the genetic diversity of *B. mori* in Colombia and offer a valuable baseline for conservation and hybrid development programs aimed at enhancing vigor and productivity.

## Introduction

*Bombyx mori* L. is an insect species belonging to the order Lepidoptera (which includes butterflies, skippers and moths). The mulberry silkworm is the cornerstone of the silk industry because of its capacity to produce high-quality silk [1,2]. For more than 5 millennia, the silkworm has served as an economic mainstay for sericulturists across Asia, with subsequent expansion to other regions of the world via the Silk Road [3–6]. Silk is a product of great importance not only for textiles because of its desirable sensory qualities [4,7] but also in biomedicine because of its biocompatibility and biodegradability [3,4,8].

*B. mori* has also been widely used as a model organism in both basic and applied research on Lepidoptera [1,4,5,9–14], a group that includes several agriculturally important species, such as pollinators and pests. Additionally, this species has been employed as a platform for heterologous protein expression in the pharmaceutical sector because of its well-studied genome [7,15–18]. Other silk properties, such as conductivity and energy storage, have enabled its use in the development of ecological and wearable electronic components [12,19–21]. The low-molecular-weight silk protein sericin has also demonstrated antioxidant, antibacterial, antiapoptotic, and antitumor properties, making it useful in the food and cosmetics industries [3,22].

The domestication process of this species from its wild ancestor, *B. mandarina,* ~ 5000 years ago resulted in more than 4,000 breeds and hybrids worldwide [2,6,7,11,14,23–26], which are typically grouped into Chinese, Japanese, European, and tropical categories [27,28]. These breeds exhibit a trade-off between productivity and resistance: temperate lines are more productive but less tolerant to infections, whereas tropical lines produce less silk but are more resistant to pathogens [12,29–31]. Additionally, lines can differ in their morphological traits, such as cocoon color and shape, larval marking, molt number, and hemolymph pigmentation, which have traditionally been used for genetic classification, identification, and breeding before the development of biochemical and molecular markers [32,33].

Owing to the morphological similarity among breeds (common yield-associated traits) despite geographic separation, inferring genetic relationships has proven difficult [33]. Molecular markers, especially microsatellites (SSRs), have therefore become key tools for understanding genetic diversity within *B. mori* germplasm collections. These markers improve strain identification, assist in linkage studies, and enhance marker-assisted selection by offering accurate and reliable information [24,34].

Parent selection is a critical step in breeding programs [35] and depends on the availability of genetic variation within the germplasm. Historically, selection has relied on economically relevant phenotypic traits, such as cocoon weight and shape or silk fiber length [14,25,30,36–39]. However, the use of molecular markers, especially simple sequence repeats (SSR) amplified via polymerase chain reaction (PCR), enables a more precise assessment of genetic diversity. Unlike dominant markers such as amplified fragment length polymorphism (AFLP) and random-amplified polymorphic DNA (RAPD), SSRs exhibit codominant inheritance, allowing heterozygotes to be distinguished from homozygotes [5,40]. Compared with RFLP markers (restricted fragment length polymorphisms), SSRs offer a greater level of polymorphism and a multiallelic nature and are not influenced by environmental conditions, making them highly reliable [28,38,41].

In Colombia, 63 silkworm breeds and one hybrid (Pílamo II) are maintained at the National Germplasm Bank housed at Universidad Tecnológica de Pereira and registered under entry number 235 in the National Registry of Biological Collections at the Alexander von Humboldt Institute of Biological Research, Colombia [42]. However, there is a notable absence of molecular and phenotypic characterization of this germplasm. The only published genetic study, by Gaviria et al. (2006) [43], evaluated 23 commercial lines via AFLP markers in combination with cocoon weight, reporting low levels of intra- and interpopulation variability. No reports exist that employ SSR markers or adapted phenotypic descriptors for Colombian breeds.

The silkworm breeds maintained in Colombia originate mainly from China and Japan and were introduced to Colombia at the beginning of the 1960s through sericulture initiatives supported by the National Federation of Coffee Growers. Further development occurred in the late 1980s and 1990s with the establishment of national hybrid lines through partnerships with Korean investors [44,45]. Since 2004, the Universidad Tecnológica de Pereira has overseen the conservation and research of this germplasm.

Given the lack of comprehensive genetic studies, the current absence of adapted phenotypic descriptors, and the limited commercial exploitation of this resource, robust characterization is urgently needed. These findings support germplasm conservation, allow the identification of valuable traits, and provide a foundation for genetic improvement programs, including hybrid development for pathogen resistance and silk quality enhancement.

Therefore, this study aimed to characterize the genetic and morphological variability of silkworm lines maintained in Colombia. This was achieved by estimating allele frequencies and genetic diversity parameters via SSR markers, evaluating phenotypic traits related to morphology, and analyzing the relationships between molecular and phenotypic data. The integration of both molecular and morphological analyses provides valuable baseline information to support future breeding programs and conservation strategies for the silkworm germplasm conserved in the country.

## Materials and methods

### Silkworm germplasm material

**Fig 1:**
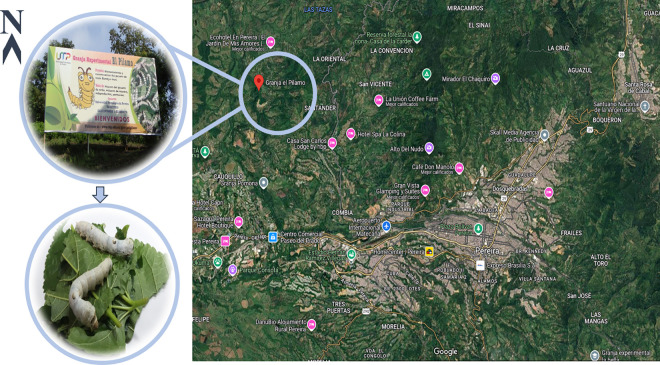
Geographical location of the *Bombyx mori* germplasm at the “El pílamo” experimental station. The farm is located in the village of La Honda, approximately 16 km from the urban area of Pereira, Risaralda, Colombia, at an elevation of 1,100 m above sea level (coordinates: 4°52’09.1”N, 75°47’36.4” W). Elaborated via Google Maps 2025. The upper inset shows the entrance to the experimental station. The lower image is illustrative and shows two individuals of the Pílamo II hybrid, one of the representative genotypes maintained in the germplasm collection.

The *Bombyx mori* germplasm evaluated in this study is maintained by the Universidad Tecnológica de Pereira at the experimental station “El Pílamo”, which is located in Pereira, Colombia. The collection included 63 purebred lines and one commercial hybrid (Pílamo II), all reared under standardized and controlled conditions (Fig 1).

The germplasm comprises 19 Chinese and 26 Japanese origin lines that were historically introduced into Colombia through national sericulture development programs beginning in the late 1960s. These lines have since been reproduced and maintained locally for several decades. Additionally, 18 noncommercial lines and Colombian hybrids developed by the Colombian Agricultural Institute (ICA) and the Sericulture Development Technology Center (CDTS) are also conserved and regularly propagated (Table 1).

**Table 1:** List of *B. mori* lines conserved by the Genetic Resource Bank of the Universidad Tecnológica de Pereira, Risaralda, Colombia. The table includes the commercial, noncommercial, and hybrid silkworm lines used in the phenotypic and genetic analyses, along with their alternative designations and country of origin. Lines labeled “NC” indicate unknown provenance (*No conocido*, in Spanish), whereas “ICA” refers to genotypes developed by the Colombian Agricultural Institute (from its acronym in Spanish).

Group	Name of the breed	Alternative name of the breed	Provenance
Commercial	CA	001	China
Commercial	CBS	002	China
Commercial	CC	003	China
Commercial	CGS	004	China
Commercial	CHS Female	005 Female	China
Commercial	CHS Male	005 Male	China
Commercial	CJ	006	China
Commercial	CLS Female	007 Female	China
Commercial	CLS Male	007 Male	China
Commercial	CTS	008	China
Commercial	NGS	009	China
Commercial	SC1	0010	China
Commercial	SC2	0011	China
Commercial	SC3	0012	China
Commercial	K01	AA	Japan
Commercial	K02	AB	Japan
Commercial	K03	AC	Japan
Commercial	K05 Female	AL Female	Japan
Commercial	K05 Male	AL Male	Japan
Commercial	K522	AG	Japan
Commercial	K10	AD	Japan
Commercial	K20	AE	Japan
Commercial	K30 Female	AM Female	Japan
Commercial	K30 Male	AM Male	Japan
Commercial	K40	AF	Japan
Commercial	SG2	AJ, HG	Japan
Commercial	SG3	AK	Japan
Commercial	KNA	AH	Japan
Commercial	KNB	AI	Japan
Hybrid	CHS X CLS K05 X K030	Pílamo II	NC
Noncommercial	450B	450B	ICA
Noncommercial	MULTILUNAR	MULTILUNAR	NC
Noncommercial	444 N	444 N	ICA
Noncommercial	NL	6A	China
Noncommercial	TA x TB	12B	Japan
Noncommercial	YP	YP	NC
Noncommercial	SG1	11B	Japan
Noncommercial	GL	3A	China
Noncommercial	LH	5A	China
Noncommercial	430 N	430 N	ICA
Noncommercial	5G3	10B	Japan
Noncommercial	Z	Z	NC
Noncommercial	K2030	7B	Japan
Noncommercial	439 N	439 N	ICA
Noncommercial	K520	4B	Japan
Noncommercial	462B	462B	ICA
Noncommercial	K5220	8B	Japan
Noncommercial	445B	445B	ICA
Noncommercial	K130	1B	Japan
Noncommercial	AKB	AKB	NC
Noncommercial	YM	YM	NC
Noncommercial	CEBRA NEGRA	CEBRA NEGRA	NC
Noncommercial	CEBRA	CEBRA	NC
Noncommercial	459 N	459 N	ICA
Noncommercial	GC3	8A	China
Noncommercial	TJ	7A	China
Noncommercial	MUTANTE	MUTANTE	NC
Noncommercial	HG	2A	China
Noncommercial	JC	1A	China
Noncommercial	TRANSPARENTE	TRANSPARENTE	NC
Noncommercial	K320	3B	Japan
Noncommercial	2G3	9B	Japan
Noncommercial	K530	5B	Japan
Noncommercial	K202	2B	Japan
Noncommercial	K1030	6B	Japan
Noncommercial	TA x KN	13B	Japan
Noncommercial	Capullo Amarillo	Capullo Amarillo	NC

The entire collection is officially registered in Colombia as a biological collection under entry number 235 in the National Register of Biological Collections (RNC), which is coordinated by the Alexander Von Humboldt Institute for Research on Biological Resources.

### Phenotypic trait analysis

Phenotypic evaluation was carried out on 63 silkworm breeds. In some of these studies, individuals were separated by sex according to parental selection schemes for commercial hybrid development. A subset of ten individuals was randomly selected per breed, and 13 phenotypic traits, including larval coloration, cocoon coloration and shape, and segmental larval markings, were recorded.

Segmental marks (observed primarily on segments 3, 5, and 8) were uniformly referred to in this study as *spiral marks* to simplify classification and analysis. These markings correspond in part to what Tanaka (1953) [46] described as *lunules* and *star spots*, although in this work, the distinction between these categories was not made for the assessment. Similarly, the presence of *ocelli* (described by Tanaka as *eye-spots*) was recorded along with associated pigmentation patterns, such as the coloration of the stripe crossing each ocellus and the interocellar region. Additional traits included facial pigmentation, prothoracic and sagittal line coloration (across anterior segments), and intersegmental pigmentation.

Commercially relevant descriptors, such as the molting pattern (moltinism), number of generations per year (voltinism), and origin (provenance) of the line, were included following the criteria reported by Kim et al*.* (2008) [14], Liu et al*.* (2010) [47], and Dalirsefat et al*.* (2007) [48].

Phenotypic traits were evaluated via a binary or categorical scoring system depending on the number of observable states. Traits such as moltinism, voltinism, larval facial coloration, cocoon shape, and the presence or absence of pigmentation features were scored as 0 or 1. Other descriptors, such as larval markings and pigmentation patterns between ocelli, were recorded via ordinal scales ranging from 0--8 (e.g., depending on the number and position of spiral marks across larval segments) or from 0--5 (e.g., according to specific color types observed).

Phenotypic evaluation was performed during the fifth-instar larval stage, which coincided with the moment of tissue sampling for molecular analysis. Cocoon traits were assessed from preexisting samples collected in a previous productivity study conducted in the laboratory (unpublished data). A complete breakdown of the scoring criteria and value assignment for each of the 13 recorded traits is provided in supplementary information S1.

### SSR marker selection and DNA extraction

Twenty-three previously reported microsatellite markers [33,49,50] were selected to assess the transferability of the molecular markers and genetic variability of the *Bombyx mori* germplasm reared in Colombia. The markers were originally developed for *B. mori* and were chosen on the basis of their levels of polymorphism and reproducibility.

Genomic DNA was extracted from 100 mg of silk gland tissue from each silkworm line via the salting-out method described by Golczer and Arrivillaga (2008) [51], with modifications implemented by researchers from the Molecular Biology and Biotechnology Laboratory at the Universidad Tecnológica de Pereira for its application in *Bombyx mori* (unpublished data).

### PCR amplification

PCRs were performed in a Veriti 96-Well Thermal Cycler (Applied Biosystems). Each 15 µL reaction mixture contained 1.5 µL of 10x reaction buffer, 0.9 µL of 25 mM MgCl_2_, 0.3 µL of 10 mM dNTPs, 0.1 µL of Taq DNA Polymerase (0.5 U/µL) (Bioline, UK), 0.3 µL of 10 µM forward and reverse primers (Table 2), 2 µL of genomic DNA (10 ng/µl), and sterile nuclease-free water to reach the final volume.

**Table 2:** Microsatellite primer information and heterozygosity and PIC values. This table includes primer sequences, microsatellite motifs, allele size ranges, allele numbers, polymorphic information content (PIC), and heterozygosity values (Ho and He) for each locus. For multilocus patterns, the results are presented separately (e.g., Locus 1, Locus 2). These markers were selected for their relevance in evaluating the genetic diversity and structure of the germplasm.

Organism	Autor	Locus symbol	Genetic sequence	Genetic motif	Allele number expected/obtained	Expected Allele size (bp)	Obtained PIC	Observed Heterozygosity (Ho)	Expected Heterozygosity (He)
*B. mori*	Li et al. 2005	F10630-F	ttttacttttcgtcgtcaccaatt	(TC)_3_TT(TC)_9_TT(TC)_2_	11/2	253-399	0,187	0.208	0.163
*B. mori*	Li et al. 2005	F10630-R	tgtaacacctacccctccgaac						
*B. mori*	Li et al. 2005	F10643-F	ttcatgtaacagtccgacaatgtatt	(TC)_8_TT(TC)_2_ATATCAT CATTCACATTCTNCTCTGG(TC)_5_	12/ Locus 1:2 Locus 2:2	144-254	Locus 1: 0.439 Locus 2: 0.376	Locus 1: 0.520 Locus 2: 0.501	Locus 1: 3.94 Locus 2: 0.328
*B. mori*	Li et al. 2005	F10643-R	gggtcggcacagcgattatt						
*B. mori*	Li et al. 2005	F10668-F	tgccttgtaggtagacgatggag	(TC)_5_C(TC)_13_C(TC)_4_C(TC)_9_	10/ Locus 1:2 Locus 2:4	233-283	Locus 1: 0.496 Locus 2: 0.435	Locus 1: 0.504 Locus 2: 0.640	Locus 1: 0.466 Locus 2: 0.389
*B. mori*	Li et al. 2005	F10668-R	agcgtaaagaccagggca						
*B. mori*	Li et al. 2005	F10659-F	ttataaaacaaagttgatcctgcca	(CA)_8_	8/2	241-261	0.095	0.075	0.090
*B. mori*	Li et al. 2005	F10659-R	gcatccgtccctcatttatcac						
*B. mori*	Li et al. 2005	F10516-F	cgactcactccttttattttattgactct	(AC)_4_AT(AC)_5_	8/4	354-378	0.543	0.367	0.455
*B. mori*	Li et al. 2005	F10516-R	cggatcgagtactgcaatgcg						
*B. mori*	Li et al. 2005	F10619-F	tatgtcacattgaggcggcg	(CA)_2_TA(CA)_9_	9/2	221-265	0,404	0.56	0.389
*B. mori*	Li et al. 2005	F10619-R	tactgcgaagtctgcgtggtc						
*B. mori*	Li et al. 2005	F10649-F	acaaacgattgaaactgaaaataga	(CA)_6_ACTCT(CA)_6_ACT(CA)_5_	7/5	246-294	0,552	0.592	0.542
*B. mori*	Li et al. 2005	F10649-R	ccaggtaaagaaagttaaccaggaag						
*B. mori*	Li et al. 2005	F10537-F	ccatttacaggctggtatccat	(CT)_15_	15/11	176-280	0.806	0.588	0.777
*B. mori*	Li et al. 2005	F10537-R	tagcgataagaccgcctattgta						
*B. mori*	Zhang et al. 2005	CA12D07R-F	gcttttgtttaactcaattatatcggg	(CA)_11_	5/ Locus 1:2 Locus 2: 4	320-390	Locus 1: 0.50 Locus 2: 0.285	Locus 1: 0.817 Locus 2: 0.787	Locus 1: 0.499 Locus 2: 0.622
*B. mori*	Zhang et al. 2005	CA12D07R-R	tccaaatgcacagatcagacctt						
*B. mori*	Zhang et al. 2005	GT18H10R-F	cgtttccgattcgttggtagatt	(GT)_12_	6/5	300-390	0,676	0.563	0.626
*B. mori*	Zhang et al. 2005	GT18H10R-R	tgtgactttgataggcgttggaa						
*B. mori*	Zhang et al. 2005	GT17F10R-F	ggctcactgaattgtatcctgctta	(GT)_8_	5/5	280-320	0.406	0.229	0.314
*B. mori*	Zhang et al. 2005	GT17F10R-R	tccaggaaagaaaattaacgcac						
*B. mori*	Zhang et al. 2005	T01CTB09R-F	acccctcggaaggtctgaata	(CT)_10_	6/ Locus 1:2 Locus 2:4	326-400	Locus 1:0.049 Locus 2:0.439	Locus 1: 0.050 Locus 2:0.458	Locus 1: 0.045 Locus 2: 0.428
*B. mori*	Zhang et al. 2005	T01CTB09R-R	gtagcatcatgtaccccgttagg						
*B. mori*	Zhang et al. 2005	T02CTD01R-F	gggtgttgccgttttaggttt	(CT)_13_	6/6	270-304	0,617	0.422	0.550
*B. mori*	Zhang et al. 2005	T02CTD01R-R	tcgttcttatccggtgtccct						
*B. mori*	Zhang et al. 2005	GT17A02R-F	tgtgctgcgtggatgtaggag	(GT)_21_	7/2	240-370	0,429	0.460	0.410
*B. mori*	Zhang et al. 2005	GT17A02R-R	gacgggcaggcggct						
*B. mori*	Zhang et al. 2005	CA16C09R-F	caagtgtgaatgtgggcgagt	(CA)_9_	5/5	230-280	0,543	0.432	0.512
*B. mori*	Zhang et al. 2005	CA16C09R-R	caatcgtgttcctgagtactttcg						
*B. mori*	Zhang et al. 2005	T01CTA07R-F	gtcagaccaaatagcggaggaa	(CT)_9_	10/ Locus 1:3 Locus 2:3	240-300	Locus 1:0.542 Locus 2: 0.508	Locus 1: 0.817 Locus 2: 1.00	Locus 1: 0.526 Locus 2: 0.508
*B. mori*	Zhang et al. 2005	T01CTA07R-R	tcgcacgccttttgttttg						
*B. mori*	Zhang et al. 2005	CA16G03R-F	acagcatccaggtccgttcc	(CA)_13_	10/ Locus 1:5 Locus 2: 2	320-410	Locus 1: 0.650 Locus 2: 0.765	Locus 1: 0.787 Locus 2: 0.781	Locus 1: 0.622 Locus 2: 0.451
*B. mori*	Zhang et al. 2005	CA16G03R-R	gccgagtaaagtatttgcgtcat						

For the primers described by Zhang et al. (2005) [50], the thermal cycling profile consisted of initial denaturation at 95 °C for 2 min; 16 cycles of 94 °C for 30 s, 63 °C to 56 °C for 1 min, and 72 °C for 1 min; 24 additional cycles of 94 °C for 30 s, 56 °C for 1 min, and 72 °C for 1 min; and a final extension step of 72 °C for 10 min, followed by a hold at 4 °C.

For the primers reported by Lie et al. (2005) [33], the cycling profile consisted of initial denaturation at 95 °C for 3 min, an annealing step at 63 °C (1 min), and 72 °C for 1 min, followed by 14 cycles of 94 °C for 30 s, 62 °C to 56 °C for 30 s, and 72 °C for 1 min. Then, 24 cycles of 94 °C for 30 s, 56 °C for 30 s, and 72 °C for 1 min were performed, with a final extension at 72 °C for 10 min and a final hold at 4 °C.

### Electrophoresis and band scoring

The PCR products were separated via 6% denaturing polyacrylamide gels (7.5 mM urea-PAGE) and visualized via silver nitrate staining [52]. A 100 bp DNA ladder (Bioline, UK) was used as a molecular weight reference to estimate the size of the amplified fragments. Microsatellite loci were scored manually via visual inspection of clear and reproducible bands. For each individual, the presence and relative position of bands on the gel were used to assign allele codes. When a single band was detected, the individual was considered homozygous (e.g., 1/1), and when two distinct bands were present, it was considered heterozygous (e.g., 1/2). These genotype assignments were used to construct a codominant matrix for downstream analyses via GenAlex and PAST software.

### Data analysis

Among the 23 SSR markers evaluated, 17 were polymorphic across the silkworm breeds analyzed (Table 2). Markers that showed monomorphic banding patterns were excluded from further analysis. Genetic diversity parameters, including allele frequency, number of alleles per locus (Na), observed heterozygosity (Ho), expected heterozygosity (He), chi-square test for Hardy‒Weinberg equilibrium, Nei’s genetic distance [53], and molecular analysis of variance (AMOVA), were calculated via GenAlex software version 6.5 [54].

The polymorphic information content (PIC) (1) for each locus was calculated via the formula described by Cordeiro, Taylor and Henry (2000) [55]:


$$\text{PIC}=1-\Sigma {P_{ij}}^{2}$$ (1)

where *P*ij is the frequency of the *j*-th allele for marker *I*, summed across all alleles.

All traits were structured into a binary or categorical checklist (see Supplementary Information S1) to ensure consistency across the dataset and facilitate statistical analysis. The resulting matrix was used to calculate the Shannon‒Weaver diversity index (H’) (1), evenness, and dominance, as well as to perform principal component analysis (PCA) and cluster analysis via the Dice similarity coefficient and the unweighted pair group method with arithmetic mean (UPGMA). Genetic relationships were further visualized via principal coordinate analysis (PCoA). These analyses were performed via PAST software (*Paleontological Statistics*) version 3.25 [56].

The Shannon‒Weaver diversity index was calculated via the following formula:


$$H\;’=-\Sigma {𝒫}_{i}\;\text{ln}\;{𝒫}_{i}\;$$ (2)

where ${𝒫}_{i}$ is the proportion of the genotypes falling into the *i*-th category of a given trait relative to the total number of genotypes evaluated.

To assess the relationship between molecular and phenotypic variation, a Mantel correlation test was conducted via GenAlex version 6.5, which is based on the Euclidean distance matrix derived from phenotypic traits and the genetic distance matrix based on SSR markers.

## Results

### Morphological diversity analysis

#### Clustering and principal component analysis.

**Fig 2:**
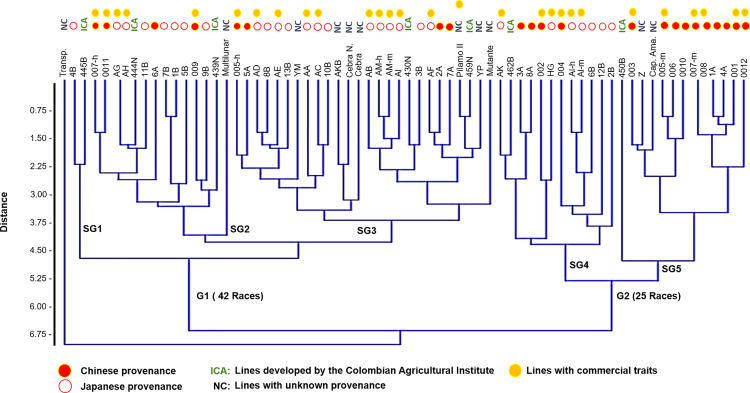
UPGMA clustering of *Bombyx mori* lines on the basis of phenotypic traits via Euclidean distance. The dendrogram shows the distribution of the 67 silkworm lines according to their morphological similarity. The colored tags above each line indicate their provenance (Chinese, Japanese, Colombian or unknown) and commercial status (commercial vs. noncommercial). Five subgroups (SG1--SG5) were identified and grouped into two major clusters (G1 and G2). These patterns suggest partial grouping by geographic origin and highlight phenotypic differentiation between breeding lines.

UPGMA cluster analysis of 13 morphological traits revealed that the silkworm germplasm was divided into two main groups and five subgroups. Group 1 contained the majority of breeds (42), whereas Group 2 comprised the remaining 25 breeds. Notably, one of the silkworm breeds (transparent) presented very low similarity, as indicated by Euclidean distances ranging from 0.0–6.75. Two potential factors could explain this grouping pattern: the first factor was related to the artificial classification system used by breeders (commercial breeds vs. noncommercial breeds), which was ruled out because of the random distribution of these categories across the dendrogram (Fig 2), and the second factor was related to the geographical origins of the breeds. When the provenance of the strains with known origins was analyzed, it became evident that the primary division observed (Groups 1 and 2) aligned with this classification, where Group 1 (G1) predominantly comprised Chinese breeds. In contrast, group 2 (G2) primarily consisted of Japanese-origin germplasms, with a few exceptions.

**Fig 3:**
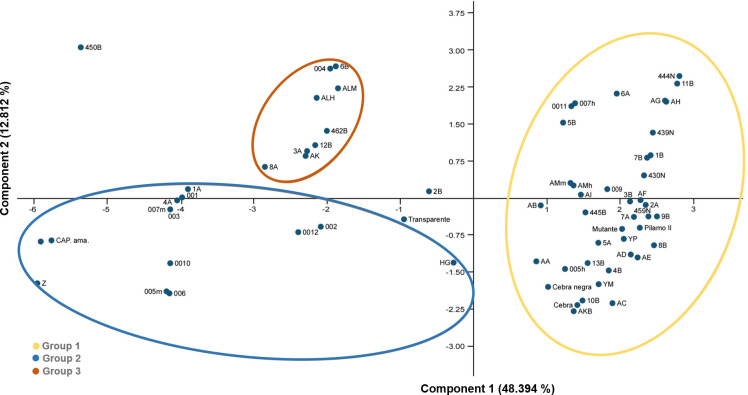
Principal component analysis (PCA) of phenotypic traits in *Bombyx mori* lines reared at the “El Pílamo” experimental station. The plot shows the distribution of 67 silkworm lines across the first two principal components (PC1 and PC2), which explain 48.39% and 12.81% of the total phenotypic variation, respectively. Three main groups (G1, G2 and G3) were identified and are indicated by colored ellipses. Three ungrouped lines (Transparente, 450B, and 2B) suggest unique morphologies or limitations of the 2D projection. This multivariate analysis confirms the grouping trends observed in the UPGMA dendrogram and reveals potential outliers with distinct phenotypic profiles.

Principal component analysis (PCA) further supported these findings, identifying three distinct clusters within the germplasm (Fig 3). Group one (G1) contained the greatest number of breeds (42 strains), whereas Groups 2 (G2) and 3 (G3) contained 13 and 9 strains, respectively. These results were consistent with the UPGMA clustering pattern. Additionally, three breeds (Transparente, 450B and 2B) did not associate with any of the three groups, suggesting that they may possess unique morphological features not captured by the primary axes of variation or that the limitation of a two-dimensional representation may obscure their true positioning, which could be better resolved in a three-dimensional analysis. The first two principal components in the PCA explained 48.39% and 12.81% of the total variation present in the germplasm, respectively.

#### Diversity indices.

Analysis of diversity indices on the basis of morphological traits revealed that 12 of the 13 evaluated characteristics contributed to explaining the diversity of the germplasm. High values for Shannon’s diversity index (H’) and evenness, along with a low dominance index, suggested substantial phenotypic variation within the collection (Table 3).

**Table 3:** Diversity indices for morphological traits evaluated in the *Bombyx mori* germplasm bank reared at the Experimental Station “El Pílamo”, Colombia. The table shows the Shannon diversity index (H’), evenness (expressed as e^H/S), and dominance values calculated for 13 phenotypic traits across 67 silkworm lines. Trait abbreviations: ORI – Provenance, VOLT – Voltinism, MOLT – Moltinism, CCOL – Cocoon Color, LAMR – Larval Markings, COSH – Cocoon Shape, COCO – Cocoon color, OCEL – Presence of Ocelli, PTLN – Prothoracic Line, OCMR – Ocellar Marking Region, SALN – Sagittal Line, FACO – Facial Coloration. These values provide quantitative evidence of morphological variability and help identify traits with greater discriminatory power across lines.

Morphological Trait	Shannon Diversity Index (H’)	Evenness (e^H/S)	Dominance
**ORIG**	3,954	0,9312	0,0204
**VOLT**	4,22	1	0,01471
**MOLT**	1,099	1	0,3333
**CCOL**	3,849	0,9208	0,02311
**LAMR**	4,057	0,9031	0,01838
**COSH**	3,555	1	0,02857
**COCO**	3,134	0,9569	0,04475
**OCEL**	3,936	0,9309	0,02077
**PTLN**	3,821	0,913	0,02313
**OCMR**	3,926	0,9391	0,02063
**SALN**	3,972	0,9649	0,01943
**FACO**	3,611	1	0,02703
**SEGM**	0,6931	1	0,5

The highest Shannon diversity index (H’) values were observed for voltinism (4.229) and larval markings (4.057), followed closely by provenance (3.954), the presence of ocelli (3.936), the interocellar marking region (3.926), and the sagittal line between thoracic segments (3.972). In contrast, the lowest H’ values were recorded for intersegmental markings (0.6931) and molting number (1.099), indicating reduced diversity in these traits across the collection.

With respect to evenness, several traits presented maximum values (1.0), including voltinism, molting number, cocoon shape, facial coloration, and intersegmental markings, reflecting a uniform distribution of these traits among the lines. The remaining traits presented moderately high evenness, ranging from 0.9031 (larval markings) to 0.9649 (sagittal line), indicating balanced trait representation.

The dominance indices were generally low for all the traits, as expected in a diverse collection. The only exception was intersegmental markings, which presented a relatively high dominance value (0.5). This finding is explained by the fact that this trait was present in only two silkworm lines (Cebra and Cebra Negra) out of the 67 evaluated, making it a rare but highly distinctive feature within the germplasm.

Among all the traits, larval marking (MRLA) emerged as the most informative, exhibiting the highest Shannon index (H’ = 4.057), the lowest dominance (0.018), and a high evenness value (0.90), highlighting its potential as a diagnostic marker for phenotypic differentiation.

### Molecular diversity analysis

#### SSR polymorphisms and marker performance.

Among the 23 microsatellite sequences evaluated, 17 presented a clear band pattern that allowed adequate analysis of the genotypes (F10601, F10630, F10643, F10668, F10659, F10516, F10619, F10649, F10537, CA12D07R, GT18H10R, GT17F10R, T01CTB09R, T02CTD01R, GT17A02R, CA16H02R, CA16C09R, T01CTA07R and CA16G03R). The markers were polymorphic in more than 86% of the loci evaluated in the four populations (Japan: 91.30%; China: 95.65%; ICA: 86.96%; and NC: 86.96%). Some markers, such as F10643, F10668, CA12D07R, T01CTB09R, T01CTA07R, and CA16G03R, revealed the presence of different loci. The number of alleles ranged from two alleles (F10619, GT17A02R, CA12D07R locus 1, CA16G03R, F10630, F10643 loci 1 and 2, T01CTB09R, F10668 loci 1 and 2, and F10659 markers) to 11 alleles (F10537 marker). The PIC value had a median of 0.46, and the effective number of alleles ranged between 1.05 alleles per locus on average (T01CTB09R-L1) and 4,496 alleles per locus on average for marker F10537.

**Fig 4:**
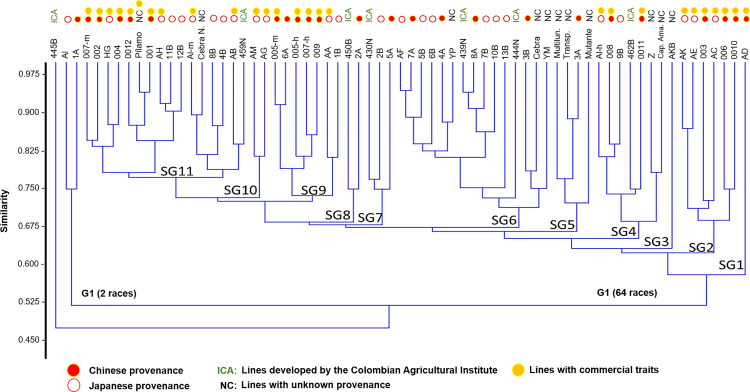
UPGMA dendrogram of the 67 *Bombyx mori* lines based on SSR marker data via the Dice similarity coefficient. Two major clusters (G1 and G2) and 11 subgroups (SG1--SG11) were identified. Line 445B appeared genetically distant from the rest of the germplasm. The colored tags represent each line’s provenance and commercial classification, as detailed in the legend of Fig 2.

**Fig 5:**
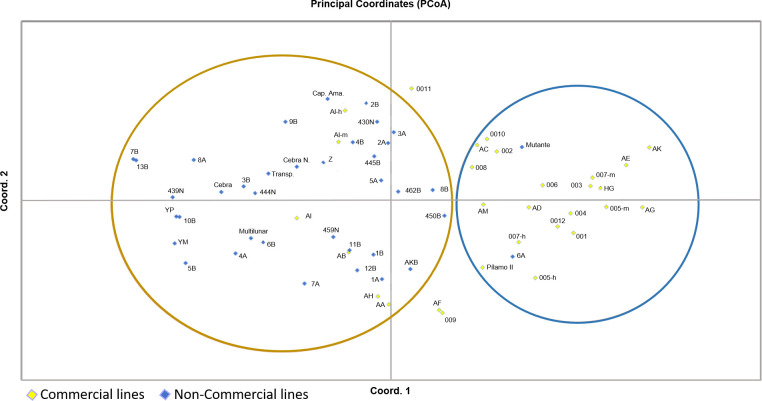
Principal coordinate analysis (PCoA) of 67 *Bombyx mori* lines on the basis of molecular data. The plot shows the spatial distributions of commercial (yellow diamonds) and noncommercial (blue diamonds) lines. Two major clusters are highlighted with colored circles, reflecting the separation pattern observed in the UPGMA dendrogram. This analysis confirms the genetic divergence between commercial and noncommercial lines and supports the potential for genotype-based grouping within the germplasm.

The UPGMA hierarchical grouping analysis, which was based on SSR marker data via the Dice similarity index, revealed two main groups and a line (445B from ICA) that was genetically distant from the rest of the germplasm. Group 1 (G1) included the closely related lines AI and 1A, whereas Group 2 (G2) contained the remaining 64 lines, which were further distributed into 11 subgroups (SG1–SG11). When these groupings are compared with conventional classification systems, such as the lines’ origin or their commercial status, no consistent clustering is observed on the basis of provenance. However, a clearer pattern emerged when lines were grouped by their commercial status: one group included all noncommercial lines, whereas the commercial lines formed two separate clusters. This distinction was also broadly supported by principal coordinate analysis (PCoA) (Figs 4 and 5).

### Genetic structure and equilibrium test

The analysis of molecular variance (AMOVA) revealed that 96% of the total genetic variation occurred within populations, whereas only 4% was attributed to differences among populations (P = 0.003; Table 4). This pattern is consistent with a relatively high level of shared genetic diversity across the evaluated silkworm lines. Additionally, the observed heterozygosity and expected heterozygosity values were generally similar across most markers, except for loci F10668, F10643, F10630, F10619, CA12D07R locus 1, CA16G03, and T01CTA07R locus 2 (see Table 2).

**Table 4:** Analysis of molecular analysis of variance (AMOVA) based on SSR markers in the *B. mori* germplasm conserved in Colombia. The table summarizes the partitioning of genetic variation within and among predefined populations, including degrees of freedom (DF), sum of squares (SS), mean squares (MS), estimated variance components, percentage of the total variance, and statistical significance. The high portion of intrapopulation variance (96%) indicates that most of the genetic diversity is distributed within the population, whereas only a small but significant portion (4%, P = 0.003) is attributable to differences among populations.

Source	DF	SS	MS	Est. Var.	% Total Var.	P
Among pop.	3	100,312	33,437	0,859	4%	**0,003**
Within Pop.	63	1270,106	20,160	20,160	96%	
**Total**	66	1370,418		21,020	100%	

A chi-square test was conducted to assess deviations from Hardy‒Weinberg equilibrium (HWE) across the different silkworm populations on the basis of their geographic origin. Among the Japanese and Chinese lines, significant and highly significant deviations from HWE were observed at several loci (Table 5), indicating that these populations are genetically unbalanced, likely due to inbreeding, genetic drift, or selection pressure affecting allele frequencies. In contrast, lines with an unknown provenance (NC “*No conocido*”) and those developed by the Colombian Agricultural Institute (ICA) showed no significant deviations at the evaluated loci, suggesting that these populations are genetically more conserved and in equilibrium under HWE assumptions.

**Table 5:** Results of the chi-square test for Hardy‒Weinberg equilibrium (HWE) in the silkworm germplasm across different SSR loci. The table shows the degrees of freedom (DF), chi-square statistics (Ji-Square), associated probability, and significance levels. Significance levels are indicated as follows: *** p < 0.001; **p < 0.01; *p < 0.05; ns = not significant.

Population	Locus	DF	Ji-Square	Probability.	P value
Japan	F10537	28	36,188	0,138	ns
Japan	F10649	6	24,990	0,000	***
Japan	CA16C09R	10	31,540	0,000	***
Japan	F10619	1	7,490	0,006	**
Japan	GT17A02R	1	0,852	0,356	ns
Japan	GT18H10R	10	34,298	0,000	***
Japan	CA12D07R-L1	1	14,410	0,000	***
Japan	CA12D07R-L2	3	12,676	0,005	**
Japan	T02CTD01R	6	20,594	0,002	**
Japan	CA16G03R-L1	3	9,523	0,023	*
Japan	CA16G03R-L2	1	8,000	0,005	**
Japan	T01CTA07R-L1	3	8,889	0,031	*
Japan	T01CTA07R-L2	1	19,000	0,000	***
Japan	F10630	Monomorphic			
Japan	F10643-L1	1	2,056	0,152	ns
Japan	F10643-L2	1	3,644	0,056	ns
Japan	T01CTB09R-L1	Monomorphic			
Japan	T01CTB09R-L2	1	3,942	0,047	*
Japan	F10668-L1	1	0,004	0,949	ns
Japan	F10668-L2	1	4,840	0,028	*
Japan	F10516	6	5,081	0,533	ns
Japan	F10659	1	3,951	0,047	*
Japan	GT17F10R	10	8,733	0,558	ns
China	F10537	21	26,707	0,181	ns
China	F10649	10	7,184	0,708	ns
China	CA16C09R	6	11,420	0,076	ns
China	F10619	1	0,444	0,505	ns
China	GT17A02R	1	0,037	0,847	ns
China	GT18H10R	3	7,147	0,067	ns
China	CA12D07R-L1	1	7,244	0,007	**
China	CA12D07R-L2	6	13,826	0,032	*
China	T02CTD01R	10	15,724	0,108	ns
China	CA16G03R-L1	3	4,105	0,250	ns
China	CA16G03R-L2	1	1,653	0,199	ns
China	T01CTA07R-L1	1	8,592	0,003	**
China	T01CTA07R-L2	3	15,000	0,002	**
China	F10630	1	0,120	0,729	ns
China	F10643-L1	1	15,278	0,000	***
China	F10643-L2	1	7,490	0,006	**
China	T01CTB09R-L1	Monomorphic			
China	T01CTB09R-L2	6	1,653	0,949	ns
China	F10668-L1	1	3,227	0,072	ns
China	F10668-L2	1	0,313	0,576	ns
China	F10516	3	11,667	0,009	**
China	F10659	1	0,123	0,725	ns
China	GT17F10R	3	12,000	0,007	**
ICA	F10537	10	18,000	0,055	ns
ICA	F10649	6	3,694	0,718	ns
ICA	CA16C09R	6	15,000	0,020	*
ICA	F10619	1	0,918	0,338	ns
ICA	GT17A02R	1	0,222	0,637	ns
ICA	GT18H10R	3	1,440	0,696	ns
ICA	CA12D07R-L1	1	0,000	1,000	ns
ICA	CA12D07R-L2	1	0,062	0,804	ns
ICA	T02CTD01R	3	2,917	0,405	ns
ICA	CA16G03R-L1	3	6,000	0,112	ns
ICA	CA16G03R-L2	1	0,222	0,637	ns
ICA	T01CTA07R-L1	1	1,440	0,230	ns
ICA	T01CTA07R-L2	1	3,000	0,083	ns
ICA	F10630	1	0,222	0,637	ns
ICA	F10643-L1	1	0,050	0,824	ns
ICA	F10643-L2	Monomorphic			
ICA	T01CTB09R-L1	1	0,062	0,804	ns
ICA	T01CTB09R-L2	3	0,750	0,861	ns
ICA	F10668-L1	1	2,000	0,157	ns
ICA	F10668-L2	1	3,000	0,083	ns
ICA	F10516	3	0,313	0,958	ns
ICA	F10659	Monomorphic			
ICA	GT17F10R	Monomorphic			
NC	F10537	28	33,778	0,208	ns
NC	F10649	6	4,247	0,643	ns
NC	CA16C09R	1	0,360	0,549	ns
NC	F10619	1	0,120	0,729	ns
NC	GT17A02R	1	0,036	0,850	ns
NC	GT18H10R	6	4,000	0,677	ns
NC	CA12D07R-L1	1	8,000	0,005	**
NC	CA12D07R-L2	3	0,194	0,978	ns
NC	T02CTD01R	6	2,063	0,914	ns
NC	CA16G03R-L1	3	12,000	0,007	**
NC	CA16G03R-L2	1	5,000	0,025	*
NC	T01CTA07R-L1	3	10,000	0,019	*
NC	T01CTA07R-L2	1	4,000	0,046	*
NC	F10630	Monomorphic			
NC	F10643-L1	1	0,090	0,764	ns
NC	F10643-L2	1	0,222	0,637	ns
NC	T01CTB09R-L1	Monomorphic			
NC	T01CTB09R-L2	6	7,556	0,273	ns
NC	F10668-L1	1	0,803	0,370	ns
NC	F10668-L2	1	0,194	0,659	ns
NC	F10516	3	7,058	0,070	ns
NC	F10659	Monomorphic			
NC	GT17F10R	1	0,120	0,729	ns

### Comparison between morphological and molecular data

#### Mantel correlation test.

**Fig 6:**
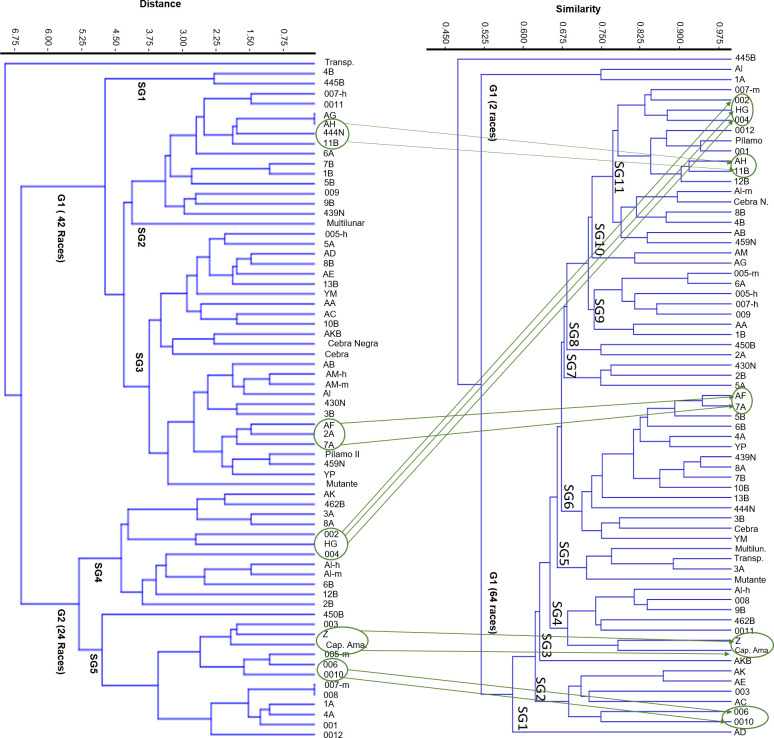
Comparative clustering of *Bombyx mori* lines on the basis of morphological and molecular data Side-by-side UPGMA dendrograms generated using phenotypic traits (left) and SSR molecular markers (right). Despite the overall divergence between classifications, some lines formed homologous clusters across both analyses, such as AH and 11B, AF and 7A, lines 002, HG and 004, Z and Capullo Amarillo, and 006 and 0010, suggesting potential conservation of trait patterns or shared ancestry.

A Mantel test comparing the morphological and molecular distance matrices revealed a very weak, nonsignificant correlation (r = 0.01414, p > 0.05), indicating minimal congruence between the two data types. These findings suggest that different evolutionary forces or selection pressures may shape phenotypic traits and molecular markers. Nonetheless, a visual comparison of UPGMA dendrograms revealed some consistent pairings, suggesting retained trait patterns or shared ancestry (Fig 6).

## Discussion

The distribution patterns of the 63 silkworm breeds, particularly those with known provenances, suggest that these phenotypic traits retain a significant portion of the ancestral genetic information from their original population. This may be attributed to backcrossing processes that preserve key morphological features while allowing adaptation to local environmental conditions [5,33]. Additionally, classification on the basis of morphological similarity offers insight into the possible origin of previously unclassified breeds within the germplasm bank. For example, the Z and Capullo Amarillo lines clustered near the Chinese-origin strain 003, whereas line 444 N showed affinity with AG and AH, both of which are of Japanese origin.

Some previously unclassified strains presented ancestral traits homologous to those of their clustered counterparts, suggesting that they may be morphologically conserved. This was particularly evident in the Multilunar, Zebra, Mutante, and 450B lines, which likely served as ancestral sources from which other phenotypes emerged in response to selective pressures. Such adaptations may lead to variation in pigmentation, which could function as aposematic warning signals against predation [**26**], increase heat absorption, and play roles in sexual selection and photoprotection via urate and melanin deposition [57,58]. Furthermore, these pigments may confer anti-ultraviolet protection and antimicrobial properties, particularly when combined with carotenoids and flavonoids derived from mulberry leaves [**59**].

The 17 microsatellite markers evaluated allowed a percentage of polymorphic loci greater than 80% to be found for the four assessed populations; in turn, six markers showed the presence of different loci, which could be associated with the polymorphic content expected by the marker, which was more remarkable for F10643 (PIC = 0.81), differing significantly from the PIC of 0.16 found by Chandrakanth et al. (2014) for this locus in 10 strains in India [**35**], and F10668 (PIC = 0.84), which aligns with values reported by the same authors. Similarly, CA12D07R (0.72), T01CTB09R (0.726) and CA16G03R (0.843) presented high polymorphism rates. In contrast, T01CTA07R presented a lower polymorphism rate (0.381) [33,49]. However, our results revealed two loci for this marker, both with PIC values > 0.50 (0.54 for locus 1 and 0.508 for locus 2), which is consistent with the PIC value of 0.88 reported by Odabaş and Cemal (2022) [**60**] in their assessment of a Turkish silkworm germplasm.

While F10668 has been described as a useful marker for distinguishing closely related silkworm lines, this study revealed lower PIC values than those reported by Li et al. (2005) [**33**] (0.496 for locus 1 and 0.435 for locus 2); for all loci, the average PIC value was 46% (range 0.05–0.81) versus 0.66 (range 0.12–0.89) for Li et al. (2005). The F10537 marker had a PIC of 0.806, and locus 2 of the CA16G03R marker had a PIC of 0.765; the first marker (F10537) presented not only a PIC of 80% but also the highest number of alleles per locus (11 alleles). Therefore, we suggest that this marker can be used to analyze the genetic diversity of silkworm germplasms raised in Colombia.

On the other hand, the 84 alleles obtained in total with a mean of 3.7 alleles/locus (range 2--11) were lower than the 188 (mean 7.2 alleles/locus, range 2--17) reported by Li et al. (2005) [**33**]; however, they do not exceed the limit established by the authors, and this phenomenon may be associated with the different biological and physiological processes to which the different ecotypes have been subjected in the studied areas, leading to molecular deletions or additions by sliding of the polymerase enzyme that would have led to changes in the frequencies of alleles.

The AMOVA test revealed that the highest genetic variance (96%) was found at the intrapopulation level and not between populations (4%) (P < 0.005); that is, the silkworm lines tended to be more homogeneous at the molecular level; however, they maintained sufficient variation to differentiate themselves from the other breeds raised by the genetic resources bank (Table 4). This finding contrasts with that reported by Gaviria et al. (2006) [**43**], who noted that the variance between silkworm populations was greater (58.57%) than the intrapopulation variance (41.43%). The low level of variance between the populations is also indicated when the observed and expected heterozygosities for the markers are evaluated.

This observed high intrapopulation genetic variability is particularly relevant for the conservation of *B. mori* in Colombia. The extent of evolutionary change in a population is proportional to the genetic diversity it harbors, and greater variability helps reduce extinction risk by increasing population fitness. This variability may guide future strategies for conservation and breeding, such as verifying the genetic resources available in the germplasm bank to avoid redundancies prior to introducing new material. Additionally, long-term conservation can be supported through modern methods, including cryopreservation of sperm, artificial insemination, induction of synthetic diapause hormones and recategorization of germplasms according to their sensitivity and storage requirements [**61**].

Slight variation is observed between both the heterozygosities of the markers evaluated in the populations, indicating that the silkworm breeds raised in this country are mainly genetically homogenizing the populations of Chinese and Japanese origin (inbreeding depression), as indicated by the chi-square test, which presents highly statistically significant differences for the evaluated microsatellite markers; that is, these two populations are genetically imbalanced, which may be associated with noncompliance with the assumptions raised by Hardy‒Weinberg, such as selective pressure, inappropriate population management, mating between these two populations by breeders for commercial purposes, inbreeding processes and a reduced size during improvement programs, which leads to a reduction in intraspecific heterozygosity and therefore a reduction in the ability of breeds to adapt to the environment [7,62].

On the other hand, it is also evident with the use of these markers that the lines whose origin is not known, such as those from the Colombian Agricultural Institute (ICA) and those that are unknown without any information (NC), are more stable at the genetic level, and they do not present statistically significant differences; therefore, they do not deviate from the Hardy‒Weinberg equilibrium (Table 5) and can be utilized to increase hybrid vigor in commercial lines while also enabling the development of more robust strains with increased resistance to diseases, better adaptability to human management, and greater productive potential. These results regarding Hardy–Weinberg equilibrium reveal contrasting patterns within the Colombian germplasm. While the Chinese and Japanese populations showed significant deviations and low heterozygosity, the ICA and NC lines maintained equilibrium and displayed higher heterozygosity. This pattern suggests greater genetic stability in the latter. In contrast, Furdui et al. (2014) reported significant deviations from HWE in cross-population analyses of *Bombyx mori* strains from Eastern Europe and Asia, supporting the presence of population structure and a possible Wahlund effect [24]. In our case, the stability observed in the ICA and NC lines may reflect limited artificial selection and reduced inbreeding, making them valuable for future breeding efforts.

At the molecular level, genetic markers, such as RAPDs [32,63] or microsatellites (SSRs) developed and analyzed by Reddy, Abraham, and Nagaraju (1999), have been implemented on silkworm lines to evaluate the genetic structure of the germplasm [50]. In both analyses, these authors managed to separate 13 silkworm accessions into two main groups, those breeds that perform diapause and those that do not perform this process; the results were similar to those obtained in this investigation when considering that commercial characteristics in general are associated with lines with diapause in the hatching process of their postures (eggs), providing better productive characteristics such as a higher body weight in caterpillars as well as more silk fibers that are longer and of higher quality [50]. On the other hand, Li et al. (2005) reported the distribution of 31 breeds of silkworms on the basis of 26 microsatellite markers, finding that they were grouped according to their origin and their voltinism, with some exceptions for both characteristics [33]; this result is explained by the genetic effect generated during the domestication and improvement processes and is similar to the selection of attributes from different lines, a finding that could explain why, genetically, the lines reared in Colombia do not show a molecular relationship according to their origin (Fig 4) but instead show a better association according to their productive characteristics (Fig 5).

This analysis enabled the identification of potential origins for lines without prior reference. For example, the Cebra Negra line shows genetic proximity to the male AL line (Japan), line 459 N to line AB (Japan), line 450B from ICA to line 2A (China), line 430 N from ICA to line 2B (Japan), YP to line 4A (China), and line 439 N to line 8A (China). Although the ICA line 444 N belongs to the same subgroup (SG6), it is the most divergent line; on the other hand, the Cebra and YM lines are genetically closer to the 3B line of Chinese origin. Although the Multilunar, Transparente, Mutante and 3A lines are in the same subgroup (SG5), the 3A and Transparente lines are much closer genetically, and these, in turn, are the same as the mutant line; thus, the latter breed has changed the least and is distant from the other three lines. Line 462B of the ICA shows closeness at the genetic level with line 0011 of Chinese origin, as indicated in Fig 6. This can be explained by the fact that silkworm breeds derive their current phenotypes from the same population of *Bombyx mandarina* silkworms, which, through artificial selection processes, generate a range of morphological traits; however, at the genotypic level, they maintain high similarity owing to the processes of genetic retrocrossing [**5**].

When comparing the dendrograms obtained in this investigation (molecular and morphological) to find races of silkworms close to each other for both analyses, it is evident that there is no complete similarity between the molecular and phenotypic data in the results of the Mantel correlation test (r = 0.01414, p > 0.05); however, the test shows a slight correlation between the data shown in Fig 6, where some breeds coincide concerning their proximity for both analyses, as occurs between lines AH and 11B; lines AF and 7A; lines 002, HG and 004; lines Z and Capullo Amarillo; and lines 006 and 0010. This may indicate that these breeds have undergone less selective pressure, and thus, their genomes have undergone fewer changes than their ancestral populations. On the other hand, the fact that the two matrices are not completely correlated may be associated with the nature of the markers, since microsatellites, which are repetitive sequences that are mainly conserved in noncoding regions, may have a lesser effect on the phenotypic traits of individuals. In contrast, environmental factors can influence the phenotype [64].

The molecular markers evaluated enabled the analysis of the germplasm distribution, revealing that the silkworm lines were divided into two groups: the first comprised two breeds, and the second comprised the remaining germplasm. In addition, the proximity between lines without any previous provenance report (ICA and NC) could be associated with other lines whose origin is known, thereby improving the understanding of this genetic resource. On the other hand, these last two populations (ICA and NC) show more significant genetic heterogeneity in addition to being in Hardy‒Weinberg equilibrium, which may be associated with a smaller number of crossing processes and with more conserved genotypes, providing a more heterogeneous resource at the genetic level that can be implemented in breeding programs where it is intended to provide genetic vigor to other breeds.

Although silkworm lines are grouped according to their origin at the morphological level, their closeness is associated with commercial characteristics at the molecular level. This is a consequence of the artificial selection processes implemented in previous genetic improvement programs, which aimed to obtain similar phenotypes aligned with specific productive interests, as supported by Xu et al. (2024), who reported that the diversity of silkworm morphology is lower in strains from China than in those from Uzbekistan, but the production characteristics are more suitable for silk utilization in Chinese strains [39]. However, such intensive selection has promoted homogenization among lines of different origins, as observed in the Chinese and Japanese populations, and has increased intrapopulation homogeneity owing to a higher expression of recessive alleles, leading to inbreeding depression [7], as indicated by the present study. Therefore, these populations require greater attention for their conservation and the development of strategies to increase genetic heterozygosity.

When both groups were compared, there was little homology between the distributions of the breeds, which was confirmed by Mantel correlation analysis (r = 0.01414, p > 0.05), except for 11 breeds (AH and 11B; the AF and 7A lines; the 002, HG and 004 lines; the Z and Capullo Amarillo lines; and the 006 and 0010 lines), which retained similarity in the vicinity for both analyses, indicating intraspecific genetic stability concerning the time that was also expressed in their phenotype, maintaining more remarkable similarity to their ancestral populations.

This study has several limitations. A known potential bias when using SSRs in *B. mori* is the low number of polymorphic SSR loci observed in lines with contrasting characteristics, as reported by Trochez-Solarte et al. (2019), who reported a range of 17–24% [5], similar to the 35% (n = 17) reported in our study. One possible explanation is size homoplasy, where alleles from different origins converge in size but differ in sequence, leading to an underestimation of actual diversity [5]. This phenomenon may partially explain the low polymorphism observed in the Chinese and Japanese lines. Therefore, future studies could benefit from complementary marker systems (e.g., SNPs or sequencing-based approaches) to provide a more accurate assessment of genetic diversity.

Additionally, this work enhances the understanding of germplasm genetic diversity by integrating complementary methodologies and combining phenotypic and molecular marker analyses. This integrative approach provides a more comprehensive and reliable assessment of the genetic variation among silkworm breeds raised in the country.

This study offers updated insights into the genetic and morphological variability of silkworm breeds raised and maintained in Colombia, many of which have not been previously documented. The findings contribute to the sericulture sector by informing conservation strategies and research initiatives aimed at genetic improvement, ultimately enhancing their potential applications in the textile, biomedical, and pharmaceutical industries.

## Conclusions

This study provides the first integrated genetic and phenotypic assessment of the Colombian *Bombyx mori* germplasm, including 63 silkworm lines. Morphological markers successfully differentiated breeds by provenance, helping to infer the origin of previously unclassified lines. SSR markers grouped the germplasm into two main clusters aligned with breeder-defined categories and revealed low heterozygosity in commercial, Chinese, and Japanese lines.

In contrast, the ICA and NC strains presented greater heterozygosity and genetic stability, making them valuable for future breeding efforts aimed at restoring hybrid vigor. The combined use of both marker systems revealed conserved phenotypic traits not captured by molecular data, which may have relevance beyond silk production, including biomedical and pharmacological applications.

Additionally, this study introduced new morphological descriptors for silkworm classification and generated updated baseline information to support conservation and genetic improvement programs in Colombia. Future research should incorporate complementary molecular approaches to overcome marker limitations and further explore the genetic potential of underutilized strains.

## Supporting information

S1 Fig Checklist used for the phenotypic characterization of the *Bombyx mori* germplasm. A visual form designed to guide the standardized observation and recording of 13 morphologically and commercially relevant traits across silkworm breeds. The traits evaluated during the fifth-instar larval stage included pigmentation patterns, larval markings, cocoon features, and breeding-related descriptors. This checklist served as a field or laboratory guide during trait assessment and preceded the numeral coding process described in the phenotypic data analysis.
(PDF)

S1 Table Scoring system used for the numerical coding of phenotypic traits in the *Bombyx mori* germplasm. Table showing the numerical values assigned to each of the 13 phenotypic traits evaluated. Traits were scored as binary (e.g., 0 = absent, 1 = present) or ordinal (e.g., 0--8 or 0--5), depending on trait variability. This codification enabled the construction of a phenotypic matrix used for the diversity analyses detailed in the Materials and Methods section.
(DOCX)
