# Supplementary material for: Genetic and morphological variation in the Colombian Bombyx mori germplasm: A first SSR-based assessment
Source: PLoS One. 2025 Aug 11;20(8):e0330183. doi: 10.1371/journal.pone.0330183 (PMC12338791; doi:10.1371/journal.pone.0330183)
Supplement: S1 Fig — A visual form designed to guide the standardized observation and recording of 13 morphologically and commercially relevant traits across silkworm breeds. The traits evaluated during the fifth-instar larval stage included pigmentation patterns, larval markings, cocoon features, and breeding-related descriptors. This checklist served as a field or laboratory guide during trait assessment and preceded the numeral coding process described in the phenotypic data analysis. (PDF) [file pone.0330183.s001.pdf]

<u>Checklist used for the phenotypic characterization of *Bombyx mori* germplasm</u>

Breed Name: ☐

Origin (Provenance) of the line: ____________________

China: ☐ Japan: ☐ ICA. (Inst. Agrop. Col.): ☐ Not known: ☐

Voltinism of the line: ____________________

Bivoltine: ☐ Tetravoltine: ☐

Moltinism of the line: ____________________

Four times (Tetramoltine): ☐ Five times (Pentamoltine): ☐

Caterpillar color: ____________________

White: ☐ Yellow: ☐ Light gray: ☐ Dark gray: ☐ Lime Yellow: ☐

Larval markings (spiral marks): ____________________

No marking (plane): ☐ Marks in seg. 3: ☐ Mark in seg. 5: ☐

Mark in seg. 8: ☐ Marks in seg. 3, 5: ☐ Marks in seg. 3 y 8: ☐

Marks in seg. 5 y 8: ☐ Marks in seg. 3, 5, 8: ☐ Marks in seg 3,4,5,6,8 y 11: ☐

Cocoon Shape: ____________________

Oval: ☐ Elliptical: ☐

Cocoon Color: ____________________

White: ☐ Yellow: ☐ Light yellow: ☐

Ocelli (*eye-spot*) dividing line: ____________________

Missing: ☐

Ocelli with a pink dividing line: ☐

Ocelli with a fluorescent orange dividing line: ☐

Ocelli with a white dividing line: ☐

Ocelli with a lime yellow dividing line: ☐

Prothoracic Line: ____________________

Missing: ☐ Pink: ☐ Reddish: ☐ Gray: ☐ Light orange: ☐

Inter Ocelli coloration: ____

Missing: ☐ Light brown: ☐ Brown: ☐

Dark Brown: ☐ Gray: ☐ Lime yellow: ☐

Sagittal line between first and second segment: ___________________________

Missing: ☐ Brown: ☐ Black: ☐ Gray: ☐

Face color of caterpillar:____

Brown: ☐ Light brown: ☐

Intersegmental pigmentation: ____

Absent: ☐ Present: ☐

S1 Fig. Checklist used for the phenotypic characterization of Bombyx mori germplasm. Visual form designed to guide the standardized observation and recording of 13 morphological and commercially relevant traits across silkworm breeds. Traits were evaluated during the fifth instar larval stage and include pigmentation patterns, larval markings, cocoon features, and breeding-related descriptors. This checklist served as the field or laboratory guide during trait assessment and preceded the numerical coding process described in phenotypic data analysis.
