# Supplementary material for: Genetic and morphological variation in the Colombian Bombyx mori germplasm: A first SSR-based assessment
Source: PLoS One. 2025 Aug 11;20(8):e0330183. doi: 10.1371/journal.pone.0330183 (PMC12338791; doi:10.1371/journal.pone.0330183)
Supplement: S1 Table — Table showing the numerical values assigned to each of the 13 phenotypic traits evaluated. Traits were scored as binary (e.g., 0 = absent, 1 = present) or ordinal (e.g., 0--8 or 0--5), depending on trait variability. This codification enabled the construction of a phenotypic matrix used for the diversity analyses detailed in the Materials and Methods section. (DOCX) [file pone.0330183.s002.docx]

**S1 Table. Scoring system used for the numerical coding of phenotypic traits in the *Bombyx mori* germplasm.** Table showing the numerical values assigned to each of the 13 phenotypic traits evaluated. Traits were scored as binary (e.g., 0 = absent, 1 = present) or ordinal (e.g., 0-8 or 0-5), depending on the trait’s variability. This codification enabled the construction of a phenotypic matrix used for diversity analyses detailed in the Materials and Methods section.

| **Trait number** | **Trait description** | **Trait value** |
| --- | --- | --- |
| **1** | **Origin (Provenance) of the line** |  |
|  | Not known | 0 |
|  | China | 1 |
|  | Japan | 2 |
|  | ICA (Instituto Colombiano Agropecuario) | 3 |
| **2** | **Voltinism of the line** |  |
|  | Bivoltine | 0 |
|  | Tetravoltine | 1 |
|  |  |  |
|  |  |  |
| **3** | **Moltinism of the line** |  |
|  | Four times (tetramoltine) | 0 |
|  | Five times (pentamoltine) | 1 |
| **4** | **Caterpillar color** |  |
|  | White | 0 |
|  | Yellow | 1 |
|  | Light gray | 2 |
|  | Dark gray | 3 |
|  | Lime yellow | 4 |
| **5** | **Larval markings (spiral marks)** |  |
|  | No markings o plane (no marks) | 0 |
|  | Larval marks on the segment 3 | 1 |
|  | Larval marks on the segment 5 | 2 |
|  | Larval marks on the segment 8 | 3 |
|  | Larval marks on the segments 3 and 5 | 4 |
|  | Larval marks on the segments 3 and 8 | 5 |
|  | Larval marks on the segments 5 and 8 | 6 |
|  | Larval marks on the segments 3, 5 and 8 | 7 |
|  | Larval marks on the segments 3, 4, 5, 6, 8 and 11 | 8 |
| **6** | **Cocoon shape** |  |
|  | Oval | 0 |
|  | Elliptical | 1 |
| **7** | **Cocoon color** |  |
|  | White | 0 |
|  | Yellow | 1 |
|  | Light yellow | 2 |
| **8** | **Ocelli (*eye-spot*) dividing line** |  |
|  | Missing | 0 |
|  | Ocelli with a pink dividing line | 1 |
|  | Ocelli with a fluorescent orange dividing line | 2 |
|  | Ocelli with a white dividing line | 3 |
|  | Ocelli with a lime yellow dividing line | 4 |
| **9** | **Prothoracic Line** |  |
|  | Missing | 0 |
|  | Pink | 1 |
|  | Reddish | 2 |
|  | Gray | 3 |
|  | Light orange |  |
| **10** | **Inter Ocelli coloration** |  |
|  | Missing | 0 |
|  | Light brown | 1 |
|  | Brown | 2 |
|  | Dark brown | 3 |
|  | Gray | 4 |
|  | Lime yellow | 5 |
| **11** | **Sagittal line between first and second segment** |  |
|  | Missing | 0 |
|  | Brown | 1 |
|  | Black | 2 |
|  | Gray | 3 |
| **12** | **Face color of caterpillar** |  |
|  | Brown | 0 |
|  | Light brown | 1 |
| **13** | **Intersegmental pigmentation** |  |
|  | Absent | 0 |
|  | Present | 1 |
